# Supplementary material for: The Systems Biology Research Tool: evolvable open-source software
Source: BMC Syst Biol. 2008 Jun 29;2:55. doi: 10.1186/1752-0509-2-55 (PMC2446383; doi:10.1186/1752-0509-2-55)
Supplement: Additional file 1 — SBRT Archive. An archive of the current version of the Systems Biology Research Tool. [file 1752-0509-2-55-S1.zip › sbrt-1.4.0/doc/developers_guide/api/sbrt/kernel/fba/comp/ReverseRxnFluxIntervalComputer.html]

ReverseRxnFluxIntervalComputer


|  |  |  |
| --- | --- | --- |
| **Overview** \| **Package** \| **Class** \| **Use** \| **Tree** \| **Deprecated** \| **Index** \| **Help** | |  |
| **PREV CLASS**   **NEXT CLASS** | **FRAMES**    **NO FRAMES**     **All Classes** |  |
| SUMMARY: NESTED \| FIELD \| CONSTR \| METHOD | DETAIL: FIELD \| CONSTR \| METHOD |  |


---


## sbrt.kernel.fba.comp Class ReverseRxnFluxIntervalComputer

```
java.lang.Object
  sbrt.kernel.fba.comp.AbstractCvComputer
      sbrt.kernel.fba.comp.AbstractFluxIntervalComputer
          sbrt.kernel.fba.comp.ReverseRxnFluxIntervalComputer
```

**All Implemented Interfaces:**: FluxCvComputer, FluxIntervalComputer

---

``` public class ReverseRxnFluxIntervalComputer extends AbstractFluxIntervalComputer ```

This class is used to compute flux intervals while constraining the fluxes of
reverse reactions to their minimum. An interval is computed by minimizing and
maximizing the flux of a given reaction. If a flux is unbounded, as they
sometimes are, Double.NEGATIVE\_INFINITY and
Double.POSITIVE\_INFINITY are used to denote the flux bounds.

**Author:**
:   This class was written and documented by Jeremiah Wright while in the
    Wagner lab.

---

**Field Summary**

**Fields inherited from class sbrt.kernel.fba.comp.AbstractFluxIntervalComputer**

`ZERO_CUTOFF`


**Constructor Summary**

`ReverseRxnFluxIntervalComputer(FbaOptComputer computer,
double zeroCutoff)`
  
          Constructs a new flux interval computer.


| **Method Summary** | |
| --- | --- |
| `protected  java.util.Map<java.lang.String,java.lang.Double>` | `getFluxMaxima(java.util.Collection<java.lang.String> rxnNames, MutableProgressMonitor progress)`             Computes and returns the maximum fluxes of the reactions with the provided names. |
| `protected  java.util.Map<java.lang.String,java.lang.Double>` | `getFluxMinima(java.util.Collection<java.lang.String> rxnNames, MutableProgressMonitor progress)`             Computes and returns the minimum fluxes of the reactions with the provided names. |
| `java.util.Map<java.lang.String,Interval>` | `getIntervals()`             Computes and returns the intervals of every flux in the fluxome used to construct this flux interval computer. |
| `java.util.Map<java.lang.String,Interval>` | `getIntervals(java.util.Collection<java.lang.String> rxnNames)`             Computes and returns the flux intervals of the reactions with the provided names. |
| `ProgressMonitor` | `getProgress()`             Returns a progress monitor for this flux interval computer. |

**Methods inherited from class sbrt.kernel.fba.comp.AbstractFluxIntervalComputer**

`getBoundedMax, getBoundedMax, getBoundedMaxWithReverseConstrained, getBoundedMin, getBoundedMin, getConstraintTolerance, getFluxConstraint, getFluxConstraints, getInterval, getIntervals, getMaximum, getMaximum, getMaxWithReverseConstrained, getMinimum, getMinimum, getReverseRxnNames, getRxnExprConstraint, getRxnExprConstraints, getZeroCutoff, restoreFluxConstraint, restoreFluxConstraints, restoreFluxConstraints, restoreRxnExprConstraint, restoreRxnExprConstraints, restoreRxnExprConstraints, setFluxConstraint, setFluxConstraints, setRxnExprConstraint, setRxnExprConstraints`

**Methods inherited from class sbrt.kernel.fba.comp.AbstractCvComputer**

`checkFluxConstraint, checkNumber, checkRxnExpr, checkRxnExprConstraint, checkRxnName, checkRxnNames, getFluxome, getRxnExpr, getRxnExpr`

**Methods inherited from class java.lang.Object**

`clone, equals, finalize, getClass, hashCode, notify, notifyAll, toString, wait, wait, wait`

**Methods inherited from interface sbrt.kernel.fba.comp.FluxCvComputer**

`getFluxome`

**Constructor Detail**

### ReverseRxnFluxIntervalComputer

```
public ReverseRxnFluxIntervalComputer(FbaOptComputer computer,
                                      double zeroCutoff)
```

:   Constructs a new flux interval computer. The fluxome of the provided
    optimization computer must be an `IrrevRxnFluxome`.

    **Parameters:**: `computer` - the computer with which all optimizations will be computed.: `zeroCutoff` - the amount by which a computed lower bound can *exceed* a computed upper bound. **Throws:**: `java.lang.ClassCastException` - if the fluxome of the provided optimization computer is not an `IrrevRxnFluxome`.


**Method Detail**

### getProgress

```
public ProgressMonitor getProgress()
```

:   Returns a progress monitor for this flux interval computer.

    :   **Returns:**: a progress monitor for this flux interval computer.

---


### getIntervals

```
public java.util.Map<java.lang.String,Interval> getIntervals()
```

:   Computes and returns the intervals of every flux in the fluxome used to
    construct this flux interval computer.

    :   **Returns:**: reaction names mapped to the computed interval of their corresponding flux.

---


### getIntervals

```
public java.util.Map<java.lang.String,Interval> getIntervals(java.util.Collection<java.lang.String> rxnNames)
```

:   Computes and returns the flux intervals of the reactions with the
    provided names.

    :   **Parameters:**: `rxnNames` - a collection of reaction names. **Returns:**: reaction names mapped to the computed interval of their corresponding flux.

---


### getFluxMinima

```
protected java.util.Map<java.lang.String,java.lang.Double> getFluxMinima(java.util.Collection<java.lang.String> rxnNames,
                                                                         MutableProgressMonitor progress)
```

:   Computes and returns the minimum fluxes of the reactions with the
    provided names. The provided progress monitor is incremented after each
    minimum flux is computed.

    :   **Parameters:**: `rxnNames` - a collection of reaction names.: `progress` - the progress monitor to be incremented. **Returns:**: reaction names mapped to their corresponding minimum flux.

---


### getFluxMaxima

```
protected java.util.Map<java.lang.String,java.lang.Double> getFluxMaxima(java.util.Collection<java.lang.String> rxnNames,
                                                                         MutableProgressMonitor progress)
```

:   Computes and returns the maximum fluxes of the reactions with the
    provided names. Each flux is maximized while the fluxes of its reverse
    reactions are constrained to their minima. The provided progress monitor
    is incremented after each maximum flux is computed.

    :   **Parameters:**: `rxnNames` - a collection of reaction names.: `progress` - the progress monitor to be incremented. **Returns:**: reaction names mapped to their corresponding maximum flux.


---


|  |  |  |
| --- | --- | --- |
| **Overview** \| **Package** \| **Class** \| **Use** \| **Tree** \| **Deprecated** \| **Index** \| **Help** | |  |
| **PREV CLASS**   **NEXT CLASS** | **FRAMES**    **NO FRAMES**     **All Classes** |  |
| SUMMARY: NESTED \| FIELD \| CONSTR \| METHOD | DETAIL: FIELD \| CONSTR \| METHOD |  |


---
